# Safety and efficacy of topical melatonin add-on in healing of diabetic foot ulcer: a double blind, randomized clinical trial

**DOI:** 10.1186/s12902-025-02057-1

**Published:** 2025-11-06

**Authors:** Fatemeh Ahmadvash, Laya Hooshmand Gharabagh, Shahram Emami, Mohammad Reza Ghafari, Faranak Ghaderi, Sina Akhavan, Ayda Esmaeili

**Affiliations:** 1 Department of Clinical Pharmacy, School of Pharmacy, Urmia University of Medical Sciences, Urmia, Iran; 2 Department of Internal Medicine, School of Medicine, Imam Khomeini Hospital, Urmia University of Medical Sciences, Urmia, Iran; 3 Department of Pharmaceutics, School of Pharmacy, Urmia University of Medical Sciences, Urmia, Iran; 4 Department of Radiology, School of Medicine, Urmia University of Medical Sciences, Imam Khomeini Hospital, Urmia, Iran; 5 Department of Pharmaceutical Biotechnology, School of Pharmacy, Urmia University of Medical Sciences, Urmia, Iran; 6 Patient Safety Research Center, Clinical Research Institute, Urmia University of Medical Sciences, Urmia, Iran

**Keywords:** Melatonin, Diabetic foot ulcer, Hydrogel, Topical

## Abstract

**Background:**

Diabetic foot ulcer (DFU) is one of the disturbing complications associated with diabetes that influence the quality of life. Melatonin with stimulating of collagen production, emerges as a promising candidate for chronic wound treatment such as diabetic foot ulcer (DFU). This clinical trial investigates the efficacy of a novel topical melatonin hydrogel in promoting the healing process of DFUs.

**Methods:**

To assess the efficacy of the topical hydrogel containing 1% melatonin on grades 1 and 2 DFUs, a double-blind, randomized, placebo-controlled trial was designed. Subjects were randomly assigned to two groups: an experimental group receiving melatonin (1 mg/cm²) and a control group receiving a placebo. The hydrogel was applied at night for two months. The number, grade, size, and healing proportion of ulcers were assessed at baseline, after one month (day 30), and at the end of the study (day 60).

**Results:**

Thirty patients with a total of fifty-three ulcers completed the study. The melatonin group demonstrated a significant decrease in ulcer number and severity grade, along with an increase in healing proportion compared to the placebo group (*p* < 0.05). The reduction in ulcer size in the melatonin group was approximately twofold greater in compare with the placebo (mean reduction: -2.7 ± 0.4 cm² vs. -1.34 ± 0.39 cm²; p-value: 0.08). Patients on melatonin with HbA1c levels ≤ 8% or with new-onset ulcers (≤ 3 weeks old) experienced statistically significant reductions in ulcer size (*p* = 0.02 and 0.04, respectively).

**Conclusions:**

Topical 1% melatonin hydrogel demonstrated efficacy and safety in reducing the number, grade, and size of DFUs when used as a complementary therapy alongside standard ulcer care. This approach may help prevent complications associated with non-healing ulcers thereby improving patients’ quality of life and functional independence.

**Trial registration:**

(Trial Id:54495; Iranian registry of clinical trial identifier code: IRCT20200220046560N1; registration date: 2021-04−09).

**Supplementary Information:**

The online version contains supplementary material available at 10.1186/s12902-025-02057-1.

## Introduction

Diabetes Mellitus is one of the most common metabolic disorders which is predicted that by 2030 about 382 million people, equivalent to 8.9% of the world’s population, globally will be affected [1]. Diabetic foot ulcer (DFU) is a common complication of diabetes, with a prevalence ranging from 4% to 10% and a higher incidence among individuals with poor glycemic control [2]. neuropathy, vasculopathy, and impaired immune function. Impairments in leg blood flow, combined with these neuropathic and vascular abnormalities, contribute to delayed wound healing in diabetic patients.

The Wagner classification system categorizes DFUs into six grades, ranging from grade 0 (intact skin) to grade 5 (foot gangrene). This classification is crucial for guiding appropriate pharmacotherapeutic interventions. The grades III to V, characterized by bone or joint infection, necessitate systemic antibiotic therapy. In contrast, grades I and II, representing more superficial ulcers, primarily require optimal glycemic control, meticulous foot and ulcer care (including dressings), weight-bearing reduction, and the use of pharmacotherapeutic agents that accelerate healing. In cases of infection in lower grade ulcers, a short course of oral antibiotics may be considered [3, 4]. Given the significant burden of DFUs, there is a pressing need for novel therapeutic agents that can effectively shorten ulcer healing times. Ongoing research is actively exploring new therapeutic strategies to promote re-epithelialization, enhance keratinocyte proliferation, and stimulate vasculogenesis, ultimately accelerating DFU healing.

Topical formulations are the most prevalent route for delivering medications to the skin. In the context of chronic wound healing, researchers often favor topical administration of pharmacologic agents with wound-healing properties, particularly in hydrogel form [5]. As highlighted by Praveen Kolimi et al., topical formulations containing growth factor derivatives have been investigated and demonstrated to be effective in promoting healing ulcers [6].

Melatonin (N-acetyl-5-methoxytryptamine), a natural hormone primarily secreted by the pineal gland, regulates circadian rhythms and exhibits diverse pharmacological effects including antioxidant properties, immune system modulation, and cardiovascular protection [7]. A 2021 meta-analysis of sixteen trials demonstrated the beneficial effects of melatonin supplementation on diabetes-related parameters such as HbA1c, fasting blood sugar, and insulin resistance index [8]. Besides that, Emerging evidence from in vitro and in vivo studies [9–11], suggests that melatonin promotes wound healing through various mechanisms, including the suppression of inflammatory markers, modulating immune cell activity (increasing Interleukin 1 (IL-1), tumor necrosis factors (TNF-α), and transforming growth factors (TGF-β)), inducing collagen formation, and acting as an angiogenic and antioxidant agent [12–14]. Vivianc et al. demonstrated improved wound healing in diabetic rats with topical application of melatonin-loaded nanoparticles [15]. Milani et al. investigated the anti-aging efficacy of a 0.1% melatonin cream applied twice daily (1 mg per 1 cm²) in women with moderate to severe skin aging, finding potential benefits for skin tone, dryness, and roughness [16]. Notably, melatonin also demonstrates neuroprotective properties in diabetic patients [17]. Shokri et al. reported that oral melatonin supplementation (6 mg/day at bedtime for 7 weeks) enhanced the effectiveness of gabapentinoid medications in reducing diabetic neuropathy pain intensity [18].

Oral melatonin administration is limited by poor and variable bioavailability due to factors such as limited gastrointestinal absorption and extensive first-pass metabolism [19]. To overcome these limitations, alternative drug delivery methods, such as transdermal formulations, have been explored [20].

Hydrogels, semi-solid dosage forms for drug delivery, can incorporate both hydrophilic and hydrophobic agents and utilize a gelling agent to transform a solution or colloidal dispersion into a consistency suitable for topical application. Compared to other topical formulations, hydrogels offer several advantages for skin application, including ease of use, removability, and a cosmetically pleasing appearance, which is a crucial factor for patient compliance [21]. Furthermore, hydrogels provide a moist environment and readily conform to the wound surface, making them particularly suitable for wound healing applications [22].

Carbomer, a well-characterized pH-responsive polymer, is a popular choice for developing controlled-release topical drug delivery systems due to its favorable properties [23, 24]. Its advantages include high viscosity in low concentration, creation of various degrees of viscosity and flow characteristics, good temperature stability and patient acceptability [25]. To the best of our knowledge, there is no published randomized, placebo-controlled clinical studies that evaluate the effects of melatonin hydrogel on diabetic foot ulcer.

Considering the established beneficial effects of melatonin on ulcer healing and the possibility of using the topical form of melatonin formulations for treating conditions such as DFU, current trial study was designed to develop and characterize a new gel formulation of melatonin, and evaluate its effects on healing process of patients with DFU.

## Materials and methods

This randomized, double-blind, placebo-controlled clinical trial enrolled patients referred to the endocrine disease clinic affiliated with Urmia University of Medical Sciences (UMSU) between December 1, 2020, and November 30, 2022. The trial was registered with the Iranian Registry of Clinical Trials under the identifier IRCT20200220046560N1 (registration Date: 2021-04−09). The Urmia University of Medical Sciences ethics committee approved the study protocol (ethics number: IR.UMSU.REC.1399.365). All participants provided written informed consent following after a thorough explanation of the study, procedures and potential risks and benefits. The study design adhered to the ethical principles outlined in the Declaration of Helsinki [26].

### Participants

Diabetic patients with foot ulcers grades 1 and 2 according to the Wagner classification [27], palpable foot pulse, and persistent s ulcers for at least two weeks [28] were eligible to participate in the study. The inclusion criteria were as followed: medical diagnosis of diabetes mellitus, patients age ≥ 18 years; Hgb A1c < 10%, ulcer size ≥ 1 cm^2^, ulcer duration of at least for two weeks, and adequate distal extremity arterial flow (ankle brachial index (ABI) >0.4). Exclusion criteria included the need for parenteral antibiotics, oral antibiotics within the past two weeks, current use of immunosuppressant medications known to interfere with wound healing, such as corticosteroids (≥ 40 mg prednisolone daily), calcineurin inhibitors, antimetabolite agents, and rituximab [29], pregnancy or lactation, intolerance or history of hypersensitivity reaction to melatonin in any dosage form, osteomyelitis, underlying skin or rheumatologic disorders that could contribute to wound development, ulcer with purulent drainage, active malignancy or ongoing chemotherapy/radiotherapy, concomitant use of topical wound healing medications, addiction to any substance, severe renal failure (glomerular filtration rate [GFR] < 15 mL/min) [30], mental or psychological conditions that could impair patient compliance.

### Sample size

Based on previous clinical trials [10, 31], a sample size of 15 participants per group was calculated to achieve 80% power with a 5% significance level (α = 0.05, β = 0.20). To account for a potential 10% dropout rate, a total of 34 participants were enrolled in the study.

### Study design

Enrolled patients were randomly assigned to either a treatment group or a control group using a two-block randomization method. All participants underwent a baseline assessment by a single radiologist, including ankle-brachial index (ABI) measurement and color Doppler ultrasonography to rule out thrombosis or severe vascular involvement. The intervention group received topical 1% melatonin gel applied nightly for 60 days, concurrent with other standard management practices including local daily foot ulcer care and mechanical off-loading. In the control group, received a placebo gel with the identical physical characteristics. The amount of placebo or melatonin gel was determined based on the ulcer size, with 1 mg of melatonin equivalent to 0.1 mL of gel per 1 cm² of wound surface area. Both the melatonin and placebo gels were stored in a refrigerator (2–8 °C). Participants received new, freshly prepared gel every 30 days.

A follow-up schedule was implemented to monitor patient progress and wound healing. Patients were visited at the clinic on three occasions: at baseline (study entry), day 30, and day 60. At each visit, wound assessment and photography were conducted. ImageJ software, a recognized tool for rapid, accurate, and reliable wound surface area measurement, was used for this purpose [32]. Additionally, to ensure proper medication use, patients received a message and a follow-up phone call from the clinic at regular intervals. Text messages were sent weekly, and phone calls were made biweekly. Laboratory testing was also conducted to assess wound healing status. Blood samples were collected and analyzed for hemoglobin A1c (HbA1c), complete blood cell count (CBC), C-reactive protein (CRP), erythrocyte sedimentation rate (ESR), and lactate dehydrogenase (LDH) at baseline and study endpoint. Adherence to medication was considered acceptable if patients maintained at least 80% compliance [33].

### Preparation of topical gel

#### Materials

Carbomer, polyethylene glycol (PEG), propylene glycol (PG), methyl paraben, and propyl paraben were purchased from FARMED SHIMI (Tehran, Iran). Melatonin was produced by LUOTIAN xinpusheng pharmaceutical co. China and purchased from Razak pharma (Tehran, Iran).

#### Preformulating studies

To deliver a targeted dose of 1 mg melatonin per 0.1 ml of topical formulation for each 1 cm² of wound surface area [34], a 1% w/v melatonin gel was developed. Excipients were carefully selected based on established drug gel formulations and relevant resources, including the Handbook of Pharmaceutical Excipients and Martindale The Complete Drug Book [29, 35]. These excipients included a solvent system for melatonin powder, preservatives for antimicrobial protection, a gelling agent, and a neutralizing agent to achieve the desired semi-solid form. Given the limited aqueous solubility of melatonin, 20% PEG and 10% PG were incorporated as cosolvents to ensure sufficient drug concentration in the gel.

#### Preparation of formulation

The formulation comprised carbomer (0.5%) as the gelling agent, polyethylene glycol 400 (20%) and propylene glycol (10%) as the solvent system, methylparaben (1.8 mg/mL) and propylparaben (0.2 mg/mL) as preservatives, and sodium hydroxide solution (10%) as a neutralizing agent. Initially, were dissolved in a portion of deionized water. and after adding carbomer, it was kept in the refrigerator (2–8°^C^) overnight to hydrate the polymer. Afterwards, the mixture was subsequently stirred for 1–2 h till it got homogenized. Concurrently, melatonin was dissolved in a mixture of polyethylene glycol and propylene glycol. The carbomer-preservative mixture was combined with the melatonin solution and stirred. Deionized water was also added to achieve a final volume of 50 ml. By using suitable amount of sodium hydroxide solution to increase the pH, melatonin gel was formulated.

### Characterization of formulation

Before the clinical trial, the prepared formulation was evaluated in terms of organoleptic properties, viscosity, spread ability, pH, stability, drug release, and microbial control tests.

#### Organoleptic characteristics

The formulation was tested for texture, physical appearance, color, homogeneity, phase separation, and presence of any solid particles. Homogeneity and texture were tested by pressing small amount of the formulation between the thumb and index finger.

#### Viscosity determination

The viscosity of the melatonin gel was measured using a Fungi-lab rotational viscometer equipped with spindle L4. The measurement was conducted at room temperature, with the spindle rotating at speeds ranging from 0 to 180 rpm.

#### Spreadibility study

The spreadability of the melatonin hydrogel was evaluated using a two-glass slide method. A 500 mg sample of the gel was placed between two glass slides (15.5 cm × 15.5 cm, 174 g). A 400 g weight was applied on top for three minutes. The diameter of the resulting spread circle was measured in centimeters. This procedure was repeated three times for both the melatonin gel and commercially available topical gels, including clindamycin gel, metronidazole gel, and diclofenac gel, for comparison.

#### pH measurement

Intact skin maintains a slightly acidic pH (4–6) essential for optimal barrier function. However, wound healing disrupts this acidic environment, leading to a more alkaline pH. As the wound progresses towards healing, the pH gradually returns to its acidic state [36]. Topical formulations designed for use on both intact and wounded skin should ideally possess a pH close to neutrality (around 6–7) to minimize potential irritation. In this study, the pH of the formulated melatonin hydrogel was measured using a digital pH meter calibrated with standard buffer solutions at pH 4, 7, and 9 before each measurement. One gram of the hydrogel was dissolved in 10 ml of deionized water, and the pH of the resulting solution was measured in triplicate at room temperature.

#### Stability tests

Stability tests including centrifuge test, temperature cycle test, and drug content stability were performed on the topical gel. A sample of formulation was centrifuged at 5,000 rpm for 15 min. at 25 °C. Any phase separation or visual changes were noted. The formulated melatonin hydrogel was subjected to a thermal stress cycle to assess its stability under fluctuating temperature conditions, mimicking potential storage and transportation environments. The formulation was exposed to a sequence of 24 h at room temperature followed by 24 h under refrigeration (2–8 °C) for a total of six days. This cycle was repeated three times. UV spectra of the formulation were acquired before and after the thermal stress testing to identify any potential changes induced by the temperature variations [37]. The melatonin content in the formulated hydrogel was quantified using high-performance liquid chromatography (HPLC) at room temperature. A reverse-phase C18 column (250 mm × 4.6 mm) was employed with a mobile phase composed of methanol and deionized water (50:50 v/v) at a flow rate of 0.7 mL/min. An injection volume of 20 µL was used. The UV detector was set at 223 nm, and a calibration curve was utilized for quantification [38]. To evaluate the long-term stability of melatonin content, samples of the formulation were stored at refrigerator temperature (2–8 °C) and room temperature for two months. The melatonin content in these stored samples was then analyzed using the aforementioned HPLC method.

### In vitro release study

The in vitro releases profile of melatonin from the formulated hydrogel was determined using a Franz diffusion cell system. The experiment was conducted at 32 ± 0.5 °C, mimicking human skin temperature, under continuous stirring at 500 rpm. The diffusion cell chamber had a diameter of 15 mm and a volume of 18 ml. Phosphate buffer (pH 6.4) was employed as the receptor medium, simulating physiological conditions.

Two hundred microliters (µL) of the formulated hydrogel, containing a total of 2 mg melatonin, was applied onto a cellulose membrane with a 12 kDa molecular weight cutoff. Samples were collected from the receptor compartment at predetermined time points over a six-hour period. To maintain sink conditions, each withdrawn sample was replaced with an equal volume of fresh phosphate buffer. The melatonin concentration in each sample was quantified using UV spectrophotometry at a wavelength of 310 nm, with a pre-established calibration curve for accurate measurement. This experiment was performed in triplicate for both the optimized gel formulation and a control melatonin suspension. The suspension was prepared by mixing 2 mg melatonin with 200 µL of phosphate buffer, replicating the concentration of melatonin in the formulated sample.

#### Microbial tests

Given that the melatonin gel formulation was non-sterile and intended to use on DFUs, antimicrobial effectiveness test, microbial limit testing, microbial limit testing, and microbiological examination of non-sterile products were conducted according to United State Pharmacopeia (USP).

In this test, after performing the preparation step, the growth rate of the added microorganisms in the samples, including Staphylococcus aureus, Pseudomonas aeruginosa, Candida albicans and Escherichia coli, was investigated during 28 days. For each microorganism, two samples were prepared for each microorganism. Microbial Limit Testing and Microbiological Examination of Non-sterile Products were performed to assess the formulation for potential microbial contamination during the preparation. After the preliminary testing to confirm the efficacy of the preservatives used in the formulation, the main samples were prepared and tested. According to USP guidelines, the result of this test is obtained within 3 to 5 days for aerobic microorganisms and 5 to 7 days for fungal or yeast microorganisms [39]. Two samples were considered for each type of microorganisms and in case of any microbial growth, the count and type should be determined.

### Standardization outcomes

A topical 1% melatonin gel was formulated using carbomer, PEG, PG, deionized water, methylparaben, propylparaben, and sodium hydroxide. The formulation was designed to be aesthetically pleasing with no unpleasant odor. Additionally, it was formulated to minimize the risk of skin irritation or sensitivity on intact or wounded skin. The homogeneity of the formulation, crucial for consistent drug delivery, remained unchanged after centrifugation and temperature cycling. This stability was maintained for up to two months post-formulation.

**Fig. 1:**
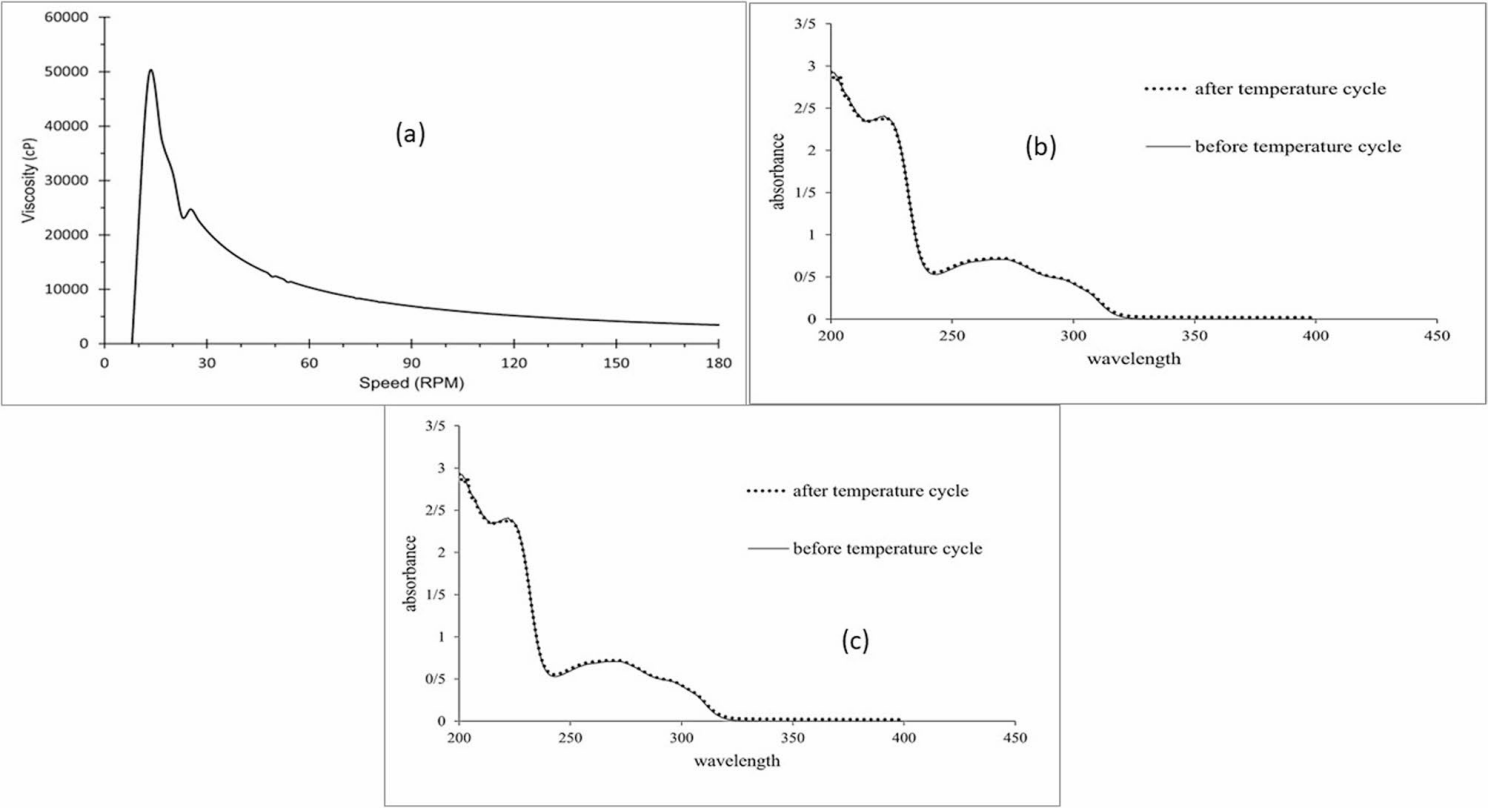
Viscosity curve (**a**), temperature cycle UV spectrums (**b**), and drug release rate (**c**) of the melatonin gel

The viscosity of the final formulation containing 0.5% carbomer ranged from 3453 to 49,000 centipoise. The Viscosity curve of formulation is illustrated in Fig. 1a. The results indicated that the formulation exhibited comparable spreadibility to commercially available gels, such as clindamycin, metronidazole, and diclofenac. The pH measurements of the prepared formulation, clindamycin topical gel and metronidazole vaginal gel were measured 6.39, 6.38, and 6.10, respectively. The results suggest that the pH of the formulation remained stable and is similar to two commonly used topical gels, clindamycin and metronidazole, making it suitable for topical skin application.

No phase separation was observed, and the formulation’s integrity remained unchanged over two months. Furthermore, no significant changes were observed in the UV spectra of formulation before and after temperature cycling indicating stability (Fig. 1b). Table 1 shows the melatonin content of the samples analyzed by HPLC. The results showed no significant melatonin content for samples stored at room temperature or in the refrigerator. Figure 1c illustrates the release profiles. The hydrogel formulation exhibited a significantly faster release rate compared to the suspension. Within four hours, 84.27% of the melatonin was released from the gel formulation, while only 44.28% was released from the suspension. After two hours, the release from the gel plateaued, while the suspension release increased slightly to 44.99%. Regarding microbial evaluation, no microbial growth was observed in the samples after the specified period for any microorganisms. As no microbial growth was detected, the microbiological examination of non-sterile products was not necessary. The absence of microbial growth indicates that the melatonin gel meets USP standards, a primary reference in the pharmaceutical industry.

**Table 1:** The amount of melatonin (mg/g) in one gram of prepared gel

Time (day)	Storage condition	Amount of melatonin (mean ± SD)
0	-	10.25 ± 0.09
7	Room*	10.38 ± 0.54
	Refrigerator**	10.62 ± 0.21
14	Room	10.28 ± 0.18
	Refrigerator	10.26 ± 0.15
21	Room	10.41 ± 0.27
	Refrigerator	10.38 ± 0.31
28	Room	10.42 ± 0.15
	Refrigerator	10.64 ± 0.36
56	Room	10.48 ± 0.15
	Refrigerator	10.11 ± 0.66

* Temperature range of 15 to 30 ℃

** Temperature range of 2 to 8 ℃

### Statistical analyses

The continuous variables were reported as mean ± standard deviation (SD) and the categorical variables were presented as numbers (percentage). To compare the frequency, the chi-square test and to compare the mean between the two groups, the Independent T-test were used. Mc-Nemar and ANCOVA were used to compare the changes of scar grade at different time points in each group. Freidman test was used to compare the changes of the wound area at different time points between the two groups. Data analyzed using SPSS20 software and *p*- value less than 0.05 was considered as significant level. |The ulcer healing percentage was calculated as follows:

$$\begin{aligned}&\text{Ulcer area percentage} =\\& \frac{area\ of\ the\ ulcer\ on\ day\ X}{area\ of\ ulcer\ at\ the\ bigining} \times 100\end{aligned}$$

$$\begin{aligned} &\text{Ulcer healing percentage} = 100 \\&- \text{Ulcer area percentage}\end{aligned}$$

## Results

### Patients and clinical outcomes

**Fig. 2:**
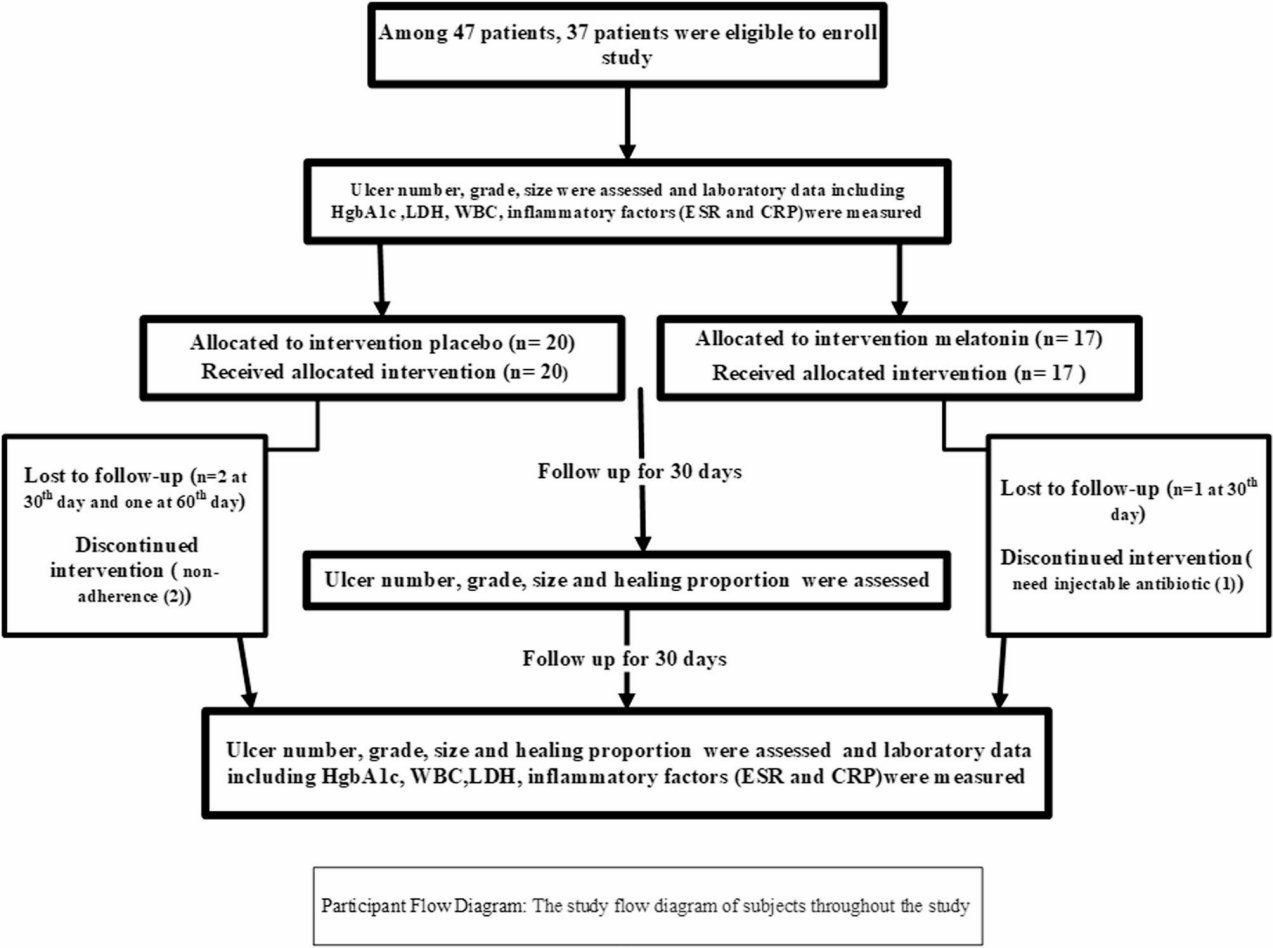
Study Flow diagram

Of 47 patients screened, 37 met the inclusion criteria and were initially enrolled. However, seven participants withdrew from the study, resulting in a final sample size of 30 patients (Fig. 2). Fifteen subjects were assigned to each group, with 33 ulcers in the melatonin group and 20 ulcers in the placebo group.

Baseline characteristics of the included patients revealed no significant differences between the groups in terms of demographic characterizations and most laboratory parameters, except for ulcer duration (Table 2). Among of the included patients, 12 patients received concurrent subcutaneous insulin and oral anti-diabetic agent s (5 patients from treatment group and 7 patients from placebo group), while18 oral anti-diabetic agents (10 in the treatment group and 8 in the placebo group). All participants were receiving moderate to high doses of atorvastatin.

**Table 2:** Patient baseline characteristics and laboratory and diabetic foot ulcer properties

		Treatment group (Melatonin) (*n* = 15)	Placebo group (Placebo) (*n* = 15)	*P*-value
Gender, No (%)	male	8 (53.3)	9 (60)	0.66^¶^
	female	7 (47.3)	6 (40)	
Age (years)		56.93 ± 7.11	58.15 ± 9.94	0.71^¥^
BMI (kg/m2)		26.24 ± 2.39	28.38 ± 5.92	0.21^¥^
Diabetes duration (years)		13.93 ± 7.42	10.92 ± 5.02	0.23^¥^
Ulcer duration (days)		72.26 ± 25.15	28.69 ± 17.03	0.004^¥^
Ulcer area (cm^2^)		3.18 ± 3.20	2.04 ± 1.51	0.29
Grade of DFU	Wegner I	12 (36.4%)	11 (55%)	0.18^¶^
	Wegner II	21 (63.6%)	9 (45%)	
Location of DFU	Toe	25	15	0.97^¶^
	Sole	8	5	
Hgb A1C %		9.33 ± 1.86	9.29 ± 1.55	0.05
FBS (mg/dl)		221.53 ± 105.52	203.69 ± 75.00	0.68
CRP (mg/L)		17.51 ± 5.77	24.12 ± 8.55	0.65
ESR (mm/hr)		40.79 ± 23.85	47.85 ± 32.65	0.82
WBC (per ml)		7.79 ± 1.80	8.12 ± 2.33	0.62
Hgb (g/dl)		12.62 ± 2.06	11.89 ± 1.86	0.34
LDH (U/L)		349.85.93 ± 100.41	350.31 ± 74.75	0.55
Vitamin D Level ng/mL		31.85 ± 18.34	30.00 ± 8.84	0.73
RI		0.71 ± 0.18	0.77 ± 0.14	0.47

*Abbreviations* Body Mass Index (BMI), Diabetic foot ulcer (DFU), Whole blood cell count (WBC), hemoglobin A1C (Hgb-A1C), Fast blood sugar (FBS), Erythrocyte sedimentation rate (ESR), C-reactive protein (CRP), Resistive index (RI), Number of patient (n)

¶: Chi-square test

¥: Mann Whitney U t-test

Table 3 shows information on the number, degree, and healing percentage of ulcers at day 30th and 60th. The number and degree of ulcers were significantly decreased in melatonin group compared with placebo group (*p* < 0.05). The healing percentage was significantly higher at day 30th and 60th in melatonin group compared to controls (*p* < 0.05). In the melatonin group, complete healing occurred in 10 out of 12 Grade I ulcers and 15 out of 21 Grade II ulcers. Only two ulcers in each Grade (I and II) showed no improvement in ulcer grade throughout the study. The change in ulcer grades in the melatonin group was statistically significant (*p* < 0.001). The placebo group showed a lower rate of complete healing. Only 5 out of 11 Grade I ulcers and 4 out of 9 grade II ulcers were completely healed. By the end of the intervention, all remaining ulcers in the placebo group had improved to Grade I. However, this change in the placebo group did not reach statistical significance (p-value = 0.07).

**Table 3:** Changes of ulcers in treatment and placebo groups

			Treatment group	Placebo group	*P*-value
Number of ulcers	Day 0		33	20	
	Day 30		18	14	0.093^¶^
	Day 60		8	10	0.03^¶^
P-value^¥^			0.001	0.063	
Grade of ulcers (percentage of all)	Day 0	1	12 (36.63%)	11 (55%)	0.18^¶^
		2	21 (63.63%)	9 (45%)	
	Day 60	0	25 (75.8%)	10 (50%)	0.003^¶^
		1	4 (12.1%)	10 (50%)	
		2	4 (12.1%)	0	
P-value^¥^			0.001	0.07	
Ulcer healing percentage	Day 30		81.76 ± 25.25	61.27 ± 39.42	0.04^€^
	Day 60		90.35 ± 19.42	88.26 ± 16.33	0.61^€^
	p-value^¥^		0.02	< 0.001	
	Day 30	toe	80.72 ± 27.91	57.48 ± 36.88	0.04^€^
		sole	87.21 ± 15.2	72.65 ± 28.31	0.28^€^
	Day 60	toe	88.21 ± 21.89	85.95 ± 18.04	0.49^€^
		sole	100.00 ± 0.00	95.21 ± 8.30	0.023^€^
	Day 30 Grade of ulcers	1	89.3 ± 14.8	68.88 ± 41.69	0.37^€^
		2	77.45 ± 29.28	50.62 ± 37.74	0.05^€^
	Day 60 Grade of ulcers	1	95.36 ± 8.58	89.12 ± 17.26	0.54^€^
		2	87.06 ± 16.84	87.37 ± 21.37	0.83^€^

¶: Comparison of numbers between two groups using Chi-square t-test

¥: Comparison of changes in each group using McNemar test (for categorical variables) or paired t-test (for continuous variables)

€: ANCOVA test: adjusted for ulcer duration, ulcer area, HA1c and RI at baseline

Melatonin treatment demonstrated significantly greater effectiveness compared to controls in reducing ulcer grade by day 60 (*p*-value = 0.003). This difference was not statistically significant at day 30 (*p*-value = 0.18). Notably, the initial ulcer grade before entering the study did not significantly influence the response to treatment in either group (*p*-value = 0.36 in melatonin, 1.0 in placebo).

**Fig. 3:**
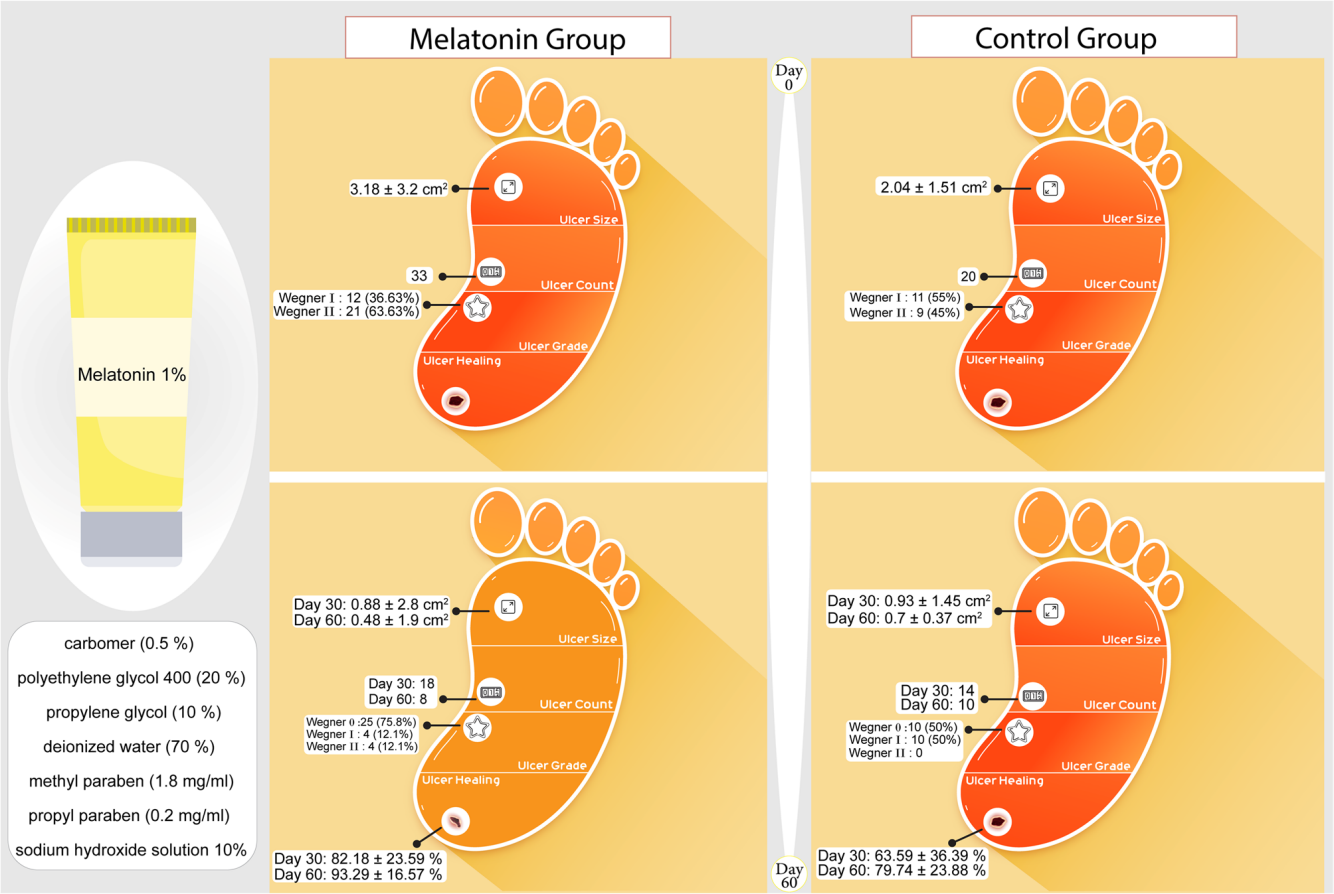
Study process and results illustration

Reductions in ulcer size were significant (*p*-value < 0.05) for both groups over the course of the trial (Table 4). However, the melatonin group demonstrated a sustained decrease in ulcer area throughout the intervention period, while the placebo group did not show a significant healing effect after day 30. Unlike the placebo group, the melatonin group exhibited statistically significant changes in ulcer size between day 30 and day 60 (p-trend < 0.001). Importantly, no patients in either group experienced an increase in ulcer size during the study. Table 4 further details the comparison of ulcer size changes between the two groups at baseline, day 30, and day 60, stratified by HbA1c levels and ulcer duration. A significant difference in the wound healing trend between the groups was observed in patients with HbA1c ≤ 8% and ulcers lasting 3 weeks or less, compared to patients with HbA1c > 8% and ulcers lasting more than 3 weeks. Melatonin treatment was more effective in reducing ulcer area for patients with new-onset ulcers and lower glycemic control (HbA1c ≤ 8%) compared to placebo (*p*-value = 0.05). By day 60, 31 out of 33 ulcers (93.9%) in the melatonin group and 15 out of 20 ulcers (75%) in the placebo group achieved at least a 50% reduction in size. All ulcers in both groups showed a minimum reduction of 25%. (Fig. 3)

**Table 4:** Changes of ulcers in treatment and placebo groups based on duration of onset of ulceration and HgbA1C

		Treatment group	within mean changes	P1	Placebo group	within mean changes	P2
		mean ± SE	mean ± SE	mean ± SE	mean ± SE		
Ulcer size (cm2)	At the beginning	3.18 ± 0.56^a^	reference	-	2.04 ± 0.32^a^	reference	-
	Day 30	0.88 ± 0.22^b^	−2.29 ± 0.35	> 0.001	0.93 ± 0.44^b^	−1.11 ± 0.25	0.001
	Day 60	0.48 ± 0.00^c^	−2.7 ± 0.4	> 0.001	0.7 ± 0.19^b^	−1.34 ± 0.39	0.001
P-trend^¶^				0.08			
Ulcer size (cm2) 8%*≥* HbA1C	At the beginning	2.79 ± 0.70^a^	reference	-	3.06 ± 1.11^a^	reference	-
	Day 30	0.39 ± 0.17^b^	−2.41 ± 0.58	0.003	2.41 ± 1. 1^a^	−0.64 ± 0.32	0.14
	Day 60	0.11 ± 0.00^b^	−2.68 ± 0.71	0.005	2.71 ± 1.15^a^	−0.35 ± 0.34	0.39
P-trend^¶^				0.02			
Ulcer size (cm2) HbA1C > 8%	At the beginning	2.75 ± 0.91^a^	reference	-	2.14 ± 0.31^a^	reference	-
	Day 30	1.38 ± 0.97^b^	−1.42 ± 0.18	0.001>	0.4 ± 0.16^b^	−1.27 ± 0.31	0.002
	Day 60	0.92 ± 0.37^b^	−1.89 ± 0.3	0.001>	0.45 ± 0.22^b^	−1.38 ± 0.31	0.001
P-trend^¶^				0.42			
Ulcer size (cm2) duration of onset of ulceration ≤ 3weeks	At the beginning	3.11 ± 0.85^a^	reference	-	2.19 ± 0.33^a^	reference	-
	Day 30	0.36 ± 0.14^b^	−2.74 ± 0.7	0.002	1.04 ± 0.76^b^	−1.15 ± 0.33	0.006
	Day 60	0.07 ± 0.0001^b^	−3.03 ± 0.78	0.001	0.29 ± 0.09^b^	−1.89 ± 0.34	0.004
P-trend^¶^				0.036			
Ulcer size (cm2) duration of onset of ulceration > 3 weeks	At the beginning	3.16 ± 0.54^a^	reference	-	1.63 ± 1.05^a^	reference	-
	Day 30	1.24 ± 0.66^b^	−1.99 ± 0.34	0.001>	0.67 ± 0.23^b^	−0.96 ± 0.27	0.04
	Day 60	0.76 ± 0.21^c^	−2.47 ± 0.43	0.001>	0.32 ± 0.09^b^	−1.30 ± 0.35	0.03
P-trend^¶^				0.28			

¶: Comparing the means between groups by Freidman test

P1: Intra-group comparison compared to the beginning (reference) in the treatment group (in each group, the same letters indicate no significant difference and different letters indicate significant difference each time versus other times) by twice comparison by Wilcoxon signed-rank test

P2: Intra-group comparison compared to the beginning (reference) in the placebo group (in each time, the same letters indicate no significant difference and different letters indicate significant difference each time versus other times) by twice comparison by Wilcoxon signed-rank test

Analysis of laboratory data revealed no significant differences between the two groups at baseline (*p*-value > 0.05). At the study endpoint, no significant changes were observed in most laboratory parameters, except for lactate dehydrogenase (LDH). LDH levels significantly decreased in the melatonin group (from 349.86 ± 93.53 to 266.00 ± 98.27; *p*-value = 0.008), while in the placebo group, LDH dropped from 350.31 ± 74.75 to 309.91 ± 61.71 (*p*-value = 0.05). Notably, both groups experienced reductions in HbA1c, ESR, CRP, and WBC at the end of the study (*p*-value ≥ 0.05).

### Safety

Throughout the study, researchers monitored all patients through bi-weekly telephone calls and weekly messages. Routine laboratory tests were performed at baseline and study endpoint. While no statistically significant changes in laboratory data were observed, it is noteworthy that the overall trends suggested improvement. Seven participants withdrew from the study for various reasons. Two patients in the placebo group demonstrated non-adherence to treatment by day 30 (with a total of 5 ulcers). One patient in the melatonin group developed a new ulcer on different body part requiring necessitating the administration of injectable antibiotics (at week 2, with 2 ulcers). Four patients (three in the placebo group with four ulcers and one in the melatonin group with two ulcers) did not return for follow-up clinic visits. Importantly, no superficial infections were reported on ulcers treated with either melatonin or placebo gels in any participant Additionally, no safety concerns regarding irritation, itching, or burning sensations were identified throughout the study.

## Discussion

DFUs are a well-recognized complication of diabetes mellitus, significantly impacting patients’ quality of life and associating with increased morbidity and mortality. Melatonin, a neurohormone with potential positive effects on the neurovascular system, immune function, and glycemic control, has been explored in pre-clinical studies for its potential role in DFU healing [20, 40]. However, to date, no published clinical investigations have evaluated the efficacy of topical melatonin hydrogel for DFU treatment. The current study demonstrated that melatonin treatment significantly reduced the number, grade and increased healing percentage of ulcers compared to controls at the end of study. Indeed, the current findings suggest that melatonin’s effectiveness in reducing the number and grade of ulcers persists until day 60, with a less pronounced difference observed at day 30 for ulcer number. Besides, the initial improvement observed in melatonin group may be attributed to its therapeutic effects, while the initial improvement in the placebo group might be attributed to the wound covering effect, which may be diminished over time.

Based on the current results, 71.42% of grade 2 ulcers exhibited complete healing in the melatonin group, only 44.44% healed in the placebo group at the study’s conclusion. Additionally, 83.33% of all grade 1 ulcers completely resolved in patients treated with melatonin at the 60 day, compared to only 45% improvement in the placebo group.

These findings demonstrate that the outcome of this study is comparable to the results of a Becaplermin 100 µg gel, an FDA-approved treatment for DFUs, which reported complete healing in 50% of patients compared to 35% in the control group [41]. Notably, in the present study, the melatonin gel achieved complete healing in 66.6% of participants.

The present study demonstrated significant reductions in ulcer size in both groups, while between-group difference was not statistically significant. Notably, the melatonin group exhibited a trend towards a greater decrease in ulcer size, with a reduction approximately twice that of the placebo group over the study period. Furthermore, melatonin group demonstrated a sustained decrease in ulcer size when comparing the squared changes in size at baseline, day 30, and day 60. In contrast, the placebo group did not display consistent effectiveness in reducing ulcer size, as evidenced by the minimal difference in ulcer size between day 30 and day 60. These findings suggest that standard wound hygiene practices may have contributed to healing in both groups during the initial 30 days. However, once a steady state was reached with regards to wound hygiene effects, the differential effectiveness of the medications became more apparent by the end of the study.

Subgroup analysis revealed that within the melatonin group, patients with HbA1c ≤ 8% and ulcer duration less than three weeks experienced significantly greater reductions in ulcer size compared to the placebo group. This finding suggests that patients with newly-onset ulcers and well-controlled glycemic may benefit more from melatonin therapy. Furthermore, the data indicates that a higher glycemic stage of diabetes and longer ulcer duration are associated with a slower healing rate. It is noteworthy that the average ulcer healing proportion in both the placebo and melatonin groups was statistically significant and generally exceeded 75%. However, the improvement observed in the melatonin group was significantly greater than that of the placebo group. These findings demonstrate that melatonin gel can be effectively used as an adjunctive therapy alongside standard ulcer hygiene practices to accelerate healing. The analysis revealed no significant influence of ulcer location or grade on the healing proportion in each group.

Baseline values of WBC, ESR, and CRP levels were assessed in all participants to determine the need for parenteral antibiotics. The non-significant changes observed in these markers throughout the study further support the initial assessment that the enrolled patients presented with stable condition with non-infectious ulcers. Lactate dehydrogenase is an enzyme released from damaged cell membranes [42]. Therefore, LDH serves as a biomarker of tissue injury and may play a crucial role in the cellular transition from inflammation to tissue remodeling [43, 44]. The significant reduction in LDH levels observed in the melatonin group suggests potential wound-healing properties of melatonin, which have been reported in other studies [45, 46].

This study has several limitations that warrant consideration when interpreting the results. The primary limitation is the relatively small sample size, which is attributable to the pilot nature of the study and the restricted eligibility criteria for participants. A larger sample size would likely enhance the generalizability of the findings and improve the study’s statistical power. Additionally, it would have been beneficial to extend the follow-up period beyond ulcer healing to assess the long-term durability of the treatment effect. This would provide valuable information regarding the sustainability of the observed improvements. Based on the findings of this study, Melatonin, with its pleiotropic properties including antioxidant activity, anti-aging, improve insulin resistance and responsiveness to anti-diabetic agents, and stimulation of collagen production, appears to be a promising therapeutic agent for promoting DFU healing. However, further research with larger sample sizes is warranted to confirm these preliminary results. Future studies should also investigate the efficacy of topical melatonin in chronic DFUs lasting at least 3 months. Additionally, exploring various topical formulations of melatonin may be beneficial to optimize delivery, wound healing properties, and potential benefits for lower limb neuropathy.

## Conclusion

This clinical trial demonstrated that daily application of a topical 1% melatonin gel (1 mg/cm²) significantly reduced the number, grade, and size of DFUs. These findings suggest that this well-tolerated hydrogel may be a promising adjunctive therapy alongside conventional ulcer hygiene practices for DFUs management. However, further research, including larger randomized controlled trials, is warranted to confirm these preliminary results and establish the long-term efficacy and safety of topical melatonin gel in the management of DFUs.

## Supplementary Information

Supplementary Material 1

Supplementary Material 2

Supplementary Material 3

## Data Availability

The data may be made available upon reasonable request from the corresponding authors; Data is provided within the manuscript or supplementary information files;
