# Supplementary figures and images for: Safety and efficacy of topical melatonin add-on in healing of diabetic foot ulcer: a double blind, randomized clinical trial
Source: BMC Endocr Disord. 2025 Nov 6;25:254. doi: 10.1186/s12902-025-02057-1 (PMC12590877; doi:10.1186/s12902-025-02057-1)

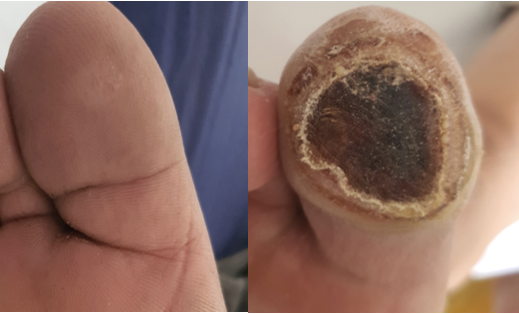


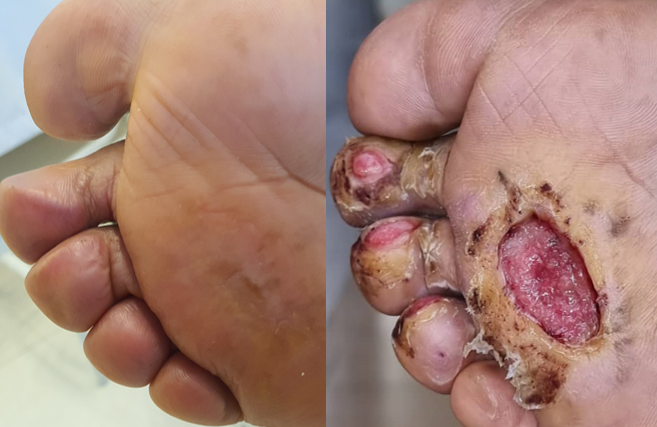


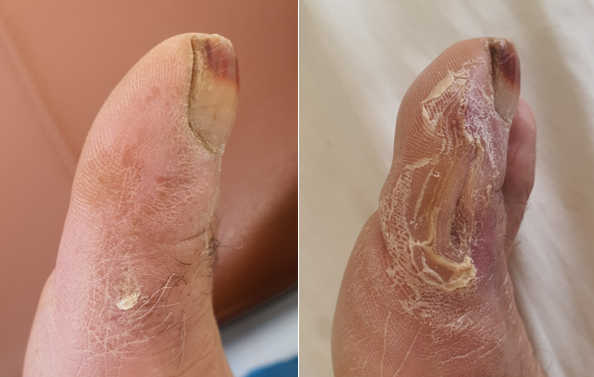

Supplement: Supplementary file 1 — Supplementary Material 1 [file 12902_2025_2057_MOESM1_ESM.docx]

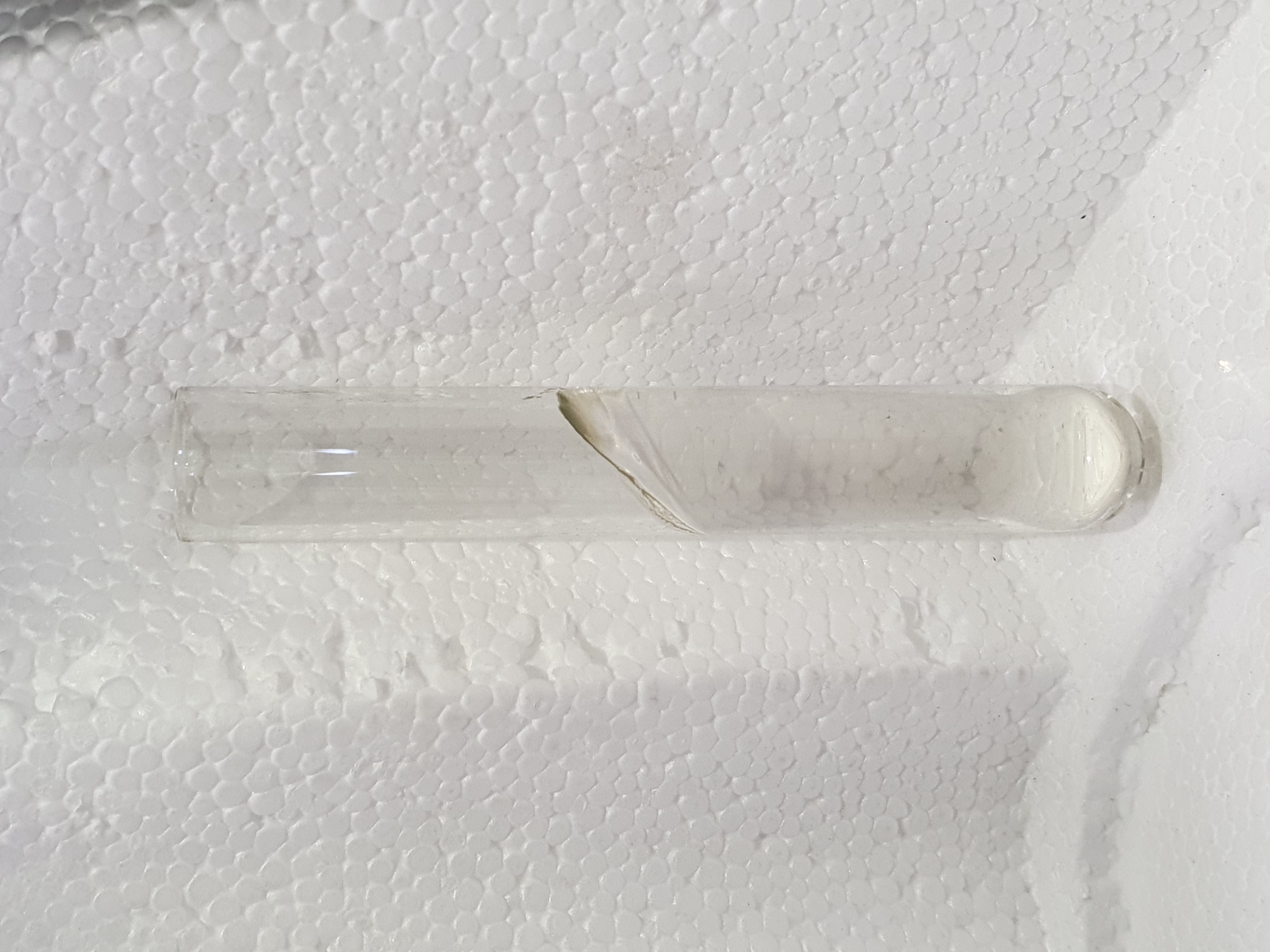

Supplement: Supplementary file 2 — Supplementary Material 2 [file 12902_2025_2057_MOESM2_ESM.jpg]

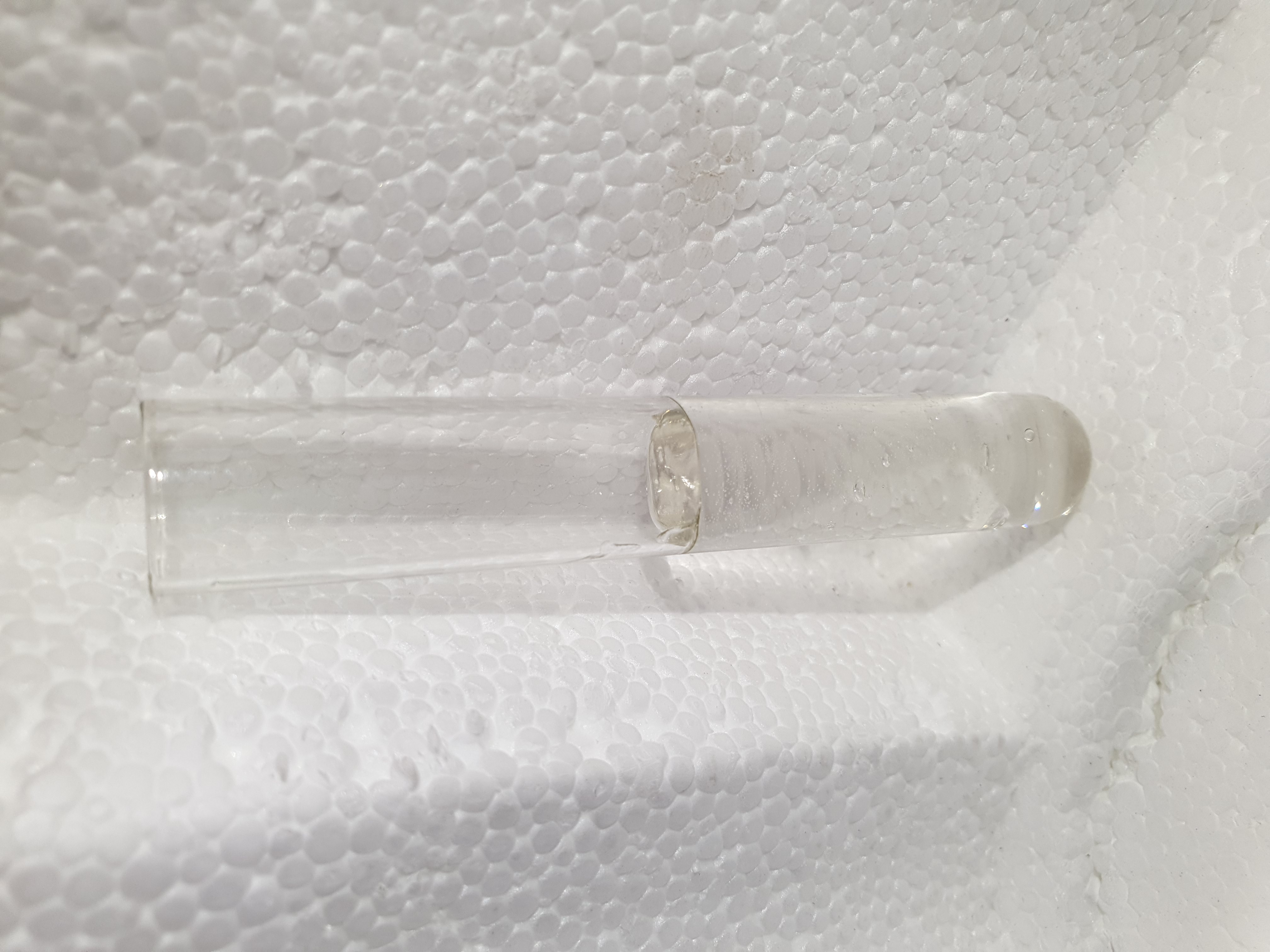

Supplement: Supplementary file 3 — Supplementary Material 3 [file 12902_2025_2057_MOESM3_ESM.jpg]
